# Correlations on Phenolic Screening Related to In Vitro and In Ovo Assessment of *Ocimum basilicum* L. Hydro-Alcoholic Extracts Used as Skin Active Ingredient

**DOI:** 10.3390/molecules25225442

**Published:** 2020-11-20

**Authors:** Alin Faur, Claudia Watz, Elena-Alina Moacă, Ştefana Avram, Florin Borcan, Iulia Pinzaru, Andrada Iftode, Mirela Nicolov, Ramona Amina Popovici, Marius Raica, Camelia A. Szuhanek, Cristina Dehelean

**Affiliations:** 1 Department of Microscopic Morphology/Histology, Angiogenesis Research Center, Faculty of Medicine, “Victor Babes” University of Medicine and Pharmacy, 2 Eftimie Murgu Square, 300041 Timisoara, Romania; alin_f32@yahoo.com (A.F.); raica@umft.ro (M.R.); 2 Department of Pharmaceutical Physics, Faculty of Pharmacy, “Victor Babes” University of Medicine and Pharmacy, 2 Eftimie Murgu Square, 300041 Timisoara, Romania; farcas.claudia@umft.ro (C.W.); nicolovmirela@gmail.com (M.N.); 3 Department of Toxicology and Drug Industry, Faculty of Pharmacy, “Victor Babes” University of Medicine and Pharmacy, 2 Eftimie Murgu Square, 300041 Timisoara, Romania; iuliapinzaru@umft.ro (I.P.); andradaiftode@umft.ro (A.I.); cadehelean@umft.ro (C.D.); 4 Department of Pharmacognosy, Faculty of Pharmacy, “Victor Babes” University of Medicine and Pharmacy, 2 Eftimie Murgu Square, 300041 Timisoara, Romania; stefana.avram@umft.ro; 5 Department of Analytical Chemistry, Faculty of Pharmacy, “Victor Babes” University of Medicine and Pharmacy, 2 Eftimie Murgu Square, 300041 Timisoara, Romania; fborcan@umft.ro; 6 Department of Management, Legislation and Communication in Dentistry, Faculty of Dentistry, “Victor Babes” University of Medicine and Pharmacy, 2 Eftimie Murgu Square, 300041 Timisoara, Romania; ramona.popovici@umft.ro; 7 Department of Orthodontics, Faculty of Dental Medicine, “Victor Babes” University of Medicine and Pharmacy, 2 Eftimie Murgu Square, 300041 Timisoara, Romania; cameliaszuhanek@umft.ro

**Keywords:** basil, DPPH, phenolic composition, cell viability, skin irritation, CAM assay

## Abstract

The current study was aimed to evaluate the phenolic composition parameters of two hydro-alcoholic extracts of *Ocimum basilicum* L. (OB) obtained from the aerial part (without leaves) and leaves, in order to determine their contribution to the antioxidant activity (AOA). Both hydro-alcoholic extracts have proven to be rich in polyphenolic compounds, flavonoids, flavonols and tannins. Therefore, the leaves’ extracts reveal an inhibition percentage of 89%, almost comparable with the standard reference (95%). To complete the toxicological profile, the study also assessed the potential cytotoxicity of basil hydro-alcoholic extracts on immortalized human keratinocytes (HaCaT), skin human fibroblasts (1BR3), mice epidermis (JB6Cl41-5a) and primary human melanocytes (HEMa) cells, correlated to A375 antitumor in vitro activity. The extracts did not induce significant cytotoxic effect on any of the selected normal cell lines but showed relevant activity on A375 cells. Considering the low values obtained regarding the irritative effects in the chorionallantoic membrane of the egg on blood vessels, we can emphasize that both extracts can be considered as biocompatible ingredients. Regarding the potential activity of hydro-alcoholic extracts on human skin, the decrease of erythema values after the application of extracts was a relevant observation which indicates the anti-inflammatory potential of *Ocimum basilicum* L.

## 1. Introduction

*Ocimum basilicum* L. (basil) belongs to the *Lamiaceae* family, genus *Ocimum*, is a medicinal plant used for many years as a culinary herb for flavoring the food products. *Ocimum basilicum* L. also known as sweet basil, basil extracts or basil based formulations, are very important due to their medicinal value, being a promising approach for the treatment of various illnesses [1,2]. Moreover, basil is used in perfumery, cosmetology, dental and oral products, sanitary due to its aroma given by the essential oils extracted which possess remarkable biological properties, such as: antimicrobial, antiviral, antioxidant, anticancer, larvicidal and antihelmintic properties [3,4,5,6,7,8,9,10]. All these pharmaceutical properties are given by the secondary metabolites, contained by Lamiaceae species. Chemical composition of *Ocimum basilicum* L. is diverse and it is characterized by a great variability in its chemotypes: linoleic acid, linalool, eugenol, methyl chavicol, methyl eugenol, geraniol, geranial, neral, methyl cinnamate, tetradecanoic acid, hexadecanoic acid, phytol, palmitin 2-mono, stigmasterol and beta-sitosterol being the major compounds [5,11,12]. *Ocimum basilicum* L. has an impressive background in traditional medicine in order to treat various diseases like headaches, coughs, fever, diarrhea, constipation, warts, worms, internal piles and kidney malfunctions [13,14]. Externally, *Ocimum bascilicum* L. oil can be used to protect from insect bites, snake bites, colds and for the treatment of rhinitis. In addition, basil treats the acne by applying its oil directly to the human skin [4]. Due to a defective epidermal permeability barrier function, the human skin becomes sensitized and its constant contact with the surrounding environment leads to an aggravation of its awareness [15]. Exposed to external factors (environmental—solar radiation, air pollution; lifestyle—cosmetic usage, smoking, alcohol; psychological—stress), sensitive skin manifests certain unpleasant sensations such as: stinging, burning, itching, pain and tingling [16,17]. After the appearance of these unpleasant sensations, the first clinical signs of sensitive skin are erythema, inflammation, edema and facial flush [18]. Nowadays, various pharmaceutical formulations can relieve the detailed signs and symptoms, but the advantages of using these products regarding the improvement or even to reduce erythema are limited. Extensive research studies are focused on the preparation and evaluation of pharmaceutical formulation based on plant phytocompounds in order to reduce erythema of sensitive skin. Wang and co-workers have demonstrated that the cream formulation based on a mixture of plant extract and essential oil (with anti-inflammatory and antiallergic properties), improved stratum corneum hydration, roughness, and erythema, without changing TEWL (transepidermal water loss). Moreover, after 28 days of treatment, the plant formulation improved texture and skin hydration [19]. In another research study, Zhang et al. have developed an herbal cream in order to assess its efficacy in reducing erythema. The authors found that the herbal formulation improves permeability barrier function as well as skin brightness and reduces the skin sensitivity and erythema in all subjects with sensitive skin [20].

The plant extracts proved to be very useful also in skin protection against solar radiation (UVA and UVB) preventing the structural degradation of elastic and collagen fibers in the skin. The UVA exposure of skin, for a long time, leads to the appearance of sags and wrinkles, so called skin aging process, termed photo aging [21]. Marwat et al. [22] insinuated that the possible anti-aging effect which *Ocimum basilicum* extract possess, is due to the antioxidant compounds like kaempferol, quercetin, isoquercetin, caffeic acid, rutin, catechin, rutinoside, apigenin, rosmarinic acid and ferulic acid. As a confirmation, Yoshikawa and co-workers [23] have evaluated the potential of *Ocimum basilicum* extract and his major component—rosmarinic acid, as anti-photoaging materials. The authors found that both compounds lead to an improvement and even prevent photoaged skin by restoring collagen fibers which are formed by chronic exposure to UVA irradiated fibroblasts. Moreover, Rasul and Akhtar [24] have prepared water in oil emulsion containing ethanol extract of basil seeds, and evaluated its effects on different parameters related to skin aging. The results obtained demonstrate that when human facial skin was treated with the as prepared emulsion based on basil ethanolic extract, the biochemical and mechanical parameters were improved.

In the present study, one of the main aims was to observe any potential activity of two hydro-alcoholic extracts, obtained from aerial part (without the leaves) and leaves of *Ocimum basilicum* L. on human skin. For this purpose were selected female volunteers with normal healthy skin as efficacy indicators and the evaluation was done by the non-invasive in vivo measuring of bio-physical parameters of the skin.

Another research study aims was the investigation of global pharmaco-toxicological profile as well as the biological activity of both hydro-alcoholic extracts. To this end, the hydro-alcoholic extracts were investigated by liquid chromatography–mass spectrometry (LC-MS) and total phenolic content (TP), total flavonoid and flavonols content (TF and TFv) and condensed tannins (TT) respectively were evaluated spectrophotometrically. The radical scavenging effect as well as the cytotoxic potential on four human and murine normal cell lines was also assessed. The results obtained were compared with the antitumor in vitro activity against the A375 cell line. In addition, the antiangiogenic potential in ovo, induced by the hydro-alcoholic basil extracts, was also investigated.

## 2. Results

### 2.1. Extraction of Ocimum basilicum L.

The yields of both hydro-alcoholic extracts were calculated as percentages in relation to the lyophilized powder of *Ocimum basilicum* L. The yield extracts were 17.2% for hydro-alcoholic extract obtained from aerial parts of basil (OBAE) and 20.3% for the hydro-alcoholic extract obtained only from leaves of basil (OBHE).

### 2.2. Phenolic Composition of Basil Hydro-Alcoholic Extracts

#### 2.2.1. Total Phenolic, Flavonoid/Flavonols Contents and Condensed Tannins

Screening intervention was designed to identify the phenolic compounds (TP), the content of flavonoids and flavonols (TF and TFv), the condensed tannins (TT) and the antioxidant capacity, exerted by the two hydro-alcoholic extracts from *Ocimum basilicum* L. aerial part and leaves. The extraction yield percentages for the hydro-alcoholic extracts from *Ocimum basilicum* L. aerial part and leaves of using 70% ethanol by maceration techniques are presented in Table 1. Likewise, in Table 1 are depicted the total phenolic, flavonoid and flavonols content, using the Folin–Ciocalteu method and aluminium colorimetric assay, respectively; as well as the condensed tannins. The highest quantity of phenols was detected in leaves extract (OBHE)—231.62 mg GAE/g dm, whereas the hydro-alcoholic extract prepared from the whole aerial part (without leaves) showed a lower content, OBAE—177.36 mg GAE/g dm.

Regarding the flavonoid and flavonols content of basil extracts, the highest quantity of flavonoids and flavonols were detected also in OBHE—48.22 mg RE/g dm and 9.43 mg RE/g dm, whereas in case of OBAE hydro-alcoholic extract, the quantities obtained were much lower. In the case of condensed tannins determination, the highest quantity was obtained the same for basil leaves extract, OBHE—11.18%, as against hydro-alcoholic extract from basil aerial part OBAE—8.27%.

#### 2.2.2. LC-MS Assessment of Hydro-Alcoholic Basil Extracts

The hydro-alcoholic extracts were subjected to LC-MS analysis by injecting a volume of 20 μL of sample and monitoring between 280 and 320 nm, the detector being set at an acquisition range from 200 to 700 nm. Identification of the chemical compounds from polyphenols class was conducted by comparing the retention times with those of the analytical standards. LC-MS analysis revealed a number of nine phenolic compounds in both hydro-alcoholic extracts of *Ocimum basilicum* L., identified as major components in the analyzed extracts. The major peaks determined are presented in Table 2, expressed in μg/g. Obtained results indicated that kaempferol was the most abundant polyphenolic quantified compound in the hydro-alcoholic extract obtained from both aerial part and leaves basil as well. Quercetin, epicathechin, rutin and resveratrol were also detected but in smaller concentrations, accompanied by traces of protocatechuic, caffeic, coumaric and gallic acid.

The OBHE hydro-alcoholic extract is richer in terms of polyphenolic compounds, with the exception of: epicathechin which has an amount of 11.043 μg/g in OBHE hydro-alcoholic extract as against 30.468 μg/g in OBAE hydro-alcoholic extract and protocatechuic acid, with an amount of 0.020 µg/g OBHE as against 0.638 µg/g OBAE.

#### 2.2.3. Total Antioxidant Activity

**Figure 1:**
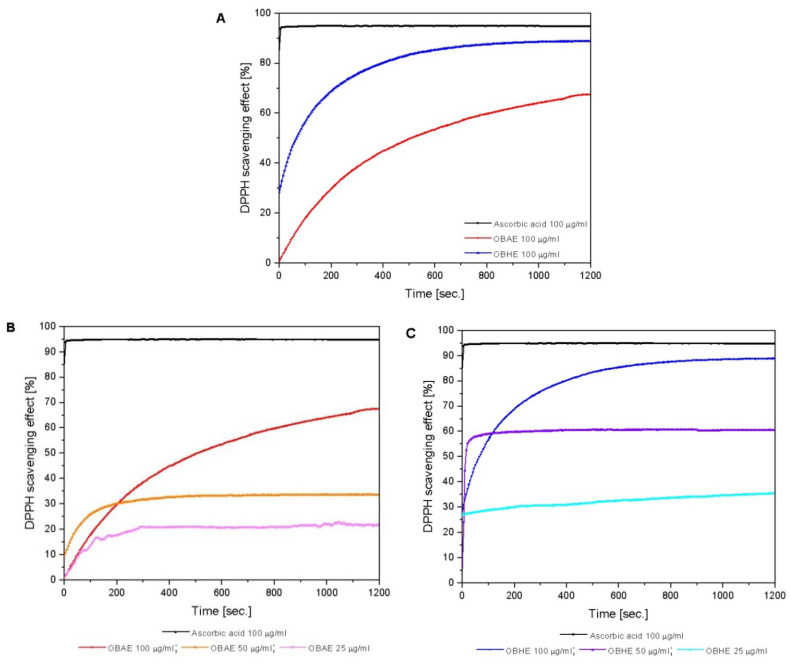
Free radical scavenging activity of hydro-alcoholoc basil extracts, (**A**)—OBAE (100 μg/mL) and OBHE (100 μg/mL) stock solution vs. ascorbic acid (AA—100 μg/mL); (**B**)—hydro-alcoholic extracts from basil aerial—OBAE (100, 50 and 25 μg/mL) vs. AA (100 μg/mL); (**C**)—hydro-alcoholic extracts from basil leaves—OBHE (100, 50 and 25 μg/mL) vs. AA (100 μg/mL).

Figure 1 revealed the DPPH (2,2-diphenyl-1-picrylhydrazyl) scavenging effect of both hydro-alcoholic basil extracts obtained from aerial part and leaves, which denoted to possess a significant activity compared to the one of ascorbic acid (AA), used as positive control. For the assessments were analyzed different concentrations of the hydro-alcoholic extracts, as follows: OBAE—100 μg/mL; 50 μg/mL and 25 μg/mL and OBHE 100 μg/mL; 50 μg/mL and 25 μg/mL respectively. From the panel (Figure 1A), it can be observed that both hydro-alcoholic extracts show high antioxidant activity. The kinetic of the reaction in the case of OBAE hydro-alcoholic extract does not reach the equilibrium even after 1200 s (20 min), instead the OBHE hydro-alcoholic extract reacts slowly with DPPH radical, scavenging the substrate in 800 s, and reaches the equilibrium after that.

Referring to the reaction kinetics of the subsequent concentrations of OBAE hydro-alcoholic extract (Figure 1B), all three concentrations react very quickly with DPPH radical, scavenging the substrate in the first 300 s; after that, the kinetic of the reaction reached equilibrium.

In the case of OBHE hydro-alcoholic extract of 50 μg/mL concentration (Figure 1C), these extracts consumed the DPPH radical very quickly, in the first 10 s, and then the equilibrium of the reaction was set. The hydro-alcoholic extract obtained from basil leaves of 25 μg/mL reacts with the DPPH radical throughout the recording time of the analysis (20 min) and the equilibrium of the reaction was not set.

In Table 3 are displayed the percent of inhibition (% inhibition) induced by standard reference (ascorbic acid in ethanol solution), *Ocimum basilicum* L. aerial part and leaves hydro-alcoholic extracts. The inhibition percent was calculated with Equation (2) described in the materials and methods part.

The extract obtained from basil leaves (OBHE) presents a percent of DPPH inhibition almost comparable with the value obtained for standard reference (89% vs. 95%). Regarding the analyzed concentrations of OBHE hydro-alcoholic extracts, the antioxidant activity (AOA) values (61% at 50 µg/mL and 36% at 25 µg/mL) were higher compared with the analyzed concentrations of OBAE hydro-alcoholic extracts (34% at 50 µg/mL and 22% at 25 µg/mL), but smaller compared with the OBAE stock solution (68% at 100 µg/mL).

All the samples analyzed have at the initial moment (time = 0 min, immediately after adding DPPH solution over the extracts) the AOA values much lower than the ones recorded at the final moment, meaning after 20 min of analysis. Based on these results, it can be concluded that the antioxidant activity of the samples was concentration-dependent.

### 2.3. Bioactivity of Basil Hydro-Alcoholic Extracts

#### Cytotoxicity of OBHE and OBAE

The cytotoxicity of *Ocimum basilicum* L. hydro-alcoholic extracts, OBHE and OBAE, was evaluated on a skin cell panel containing three human healthy cell lines: immortalized human keratinocytes (HaCaT), skin human fibroblasts (1BR3), and primary melanocytes (HEMa) and one murine healthy cell line: newborn mice epidermis (JB6 Cl 41-5a), as well as on one tumorigenic cell line—A375 skin primary achromic human melanoma cells. The method employed in this study was the consecrated MTT colorimetric test. The assessment was performed with different concentrations, 25, 50, 75, and 150 µg/mL, of OBHE and OBAE after a stimulation time of 24 h.

**Figure 2:**
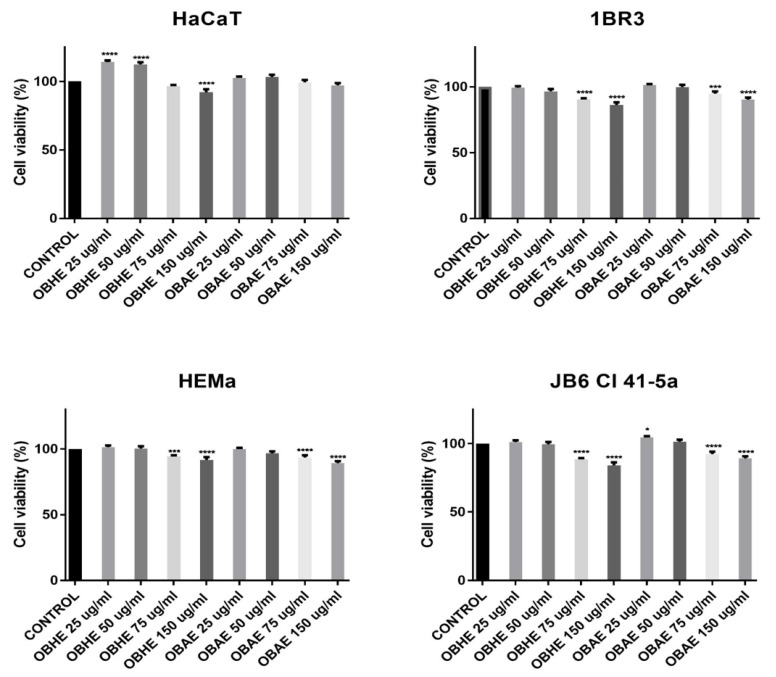
Viability percentage of HaCaT, 1BR3, HEMa and JB6Cl41-5a cells after stimulation with basil hydro-alcoholic extracts (25, 50, 75 and 150 µg/mL), at 24 h post-stimulation. The results are expressed as cell viability percentage (%) normalized to control cells. The data represent the mean value ±SD obtained from three independent experiments performed in triplicate. One-way ANOVA analysis was applied to determine the statistical differences in rapport with positive control-treated cells followed by the Tukey’s test (* *p* <0.05, *** *p* <0.001 and **** *p* <0.0001).

The results (Figure 2) indicated that test extracts, OBHE and OBAE, did not induce the important cell viability reduction of any healthy cell line in the skin cell panel. The lowest cell viability rates were recorded after exposure of HaCaT cells at 150 µg/mL of OBHE (~89% viable cells) and JB6 Cl 41-5a cells at the highest concentrations tested 75 and 150 µg/mL of OBHE (~88% and ~84%, respectively), followed by treatment of 1BR3 cell with OBHE at concentration of 150 µg/mL (~86%). A slight proliferative effect could be noticed after stimulation of HaCaT cells with OBHE at concentrations of 25 and 50 µg/mL (~115% and ~112%, respectively). While HEMa cells presented cell viability percentages above 89%, after stimulation with both test extracts, corresponding to a non-cytotoxic effect.

**Figure 3:**
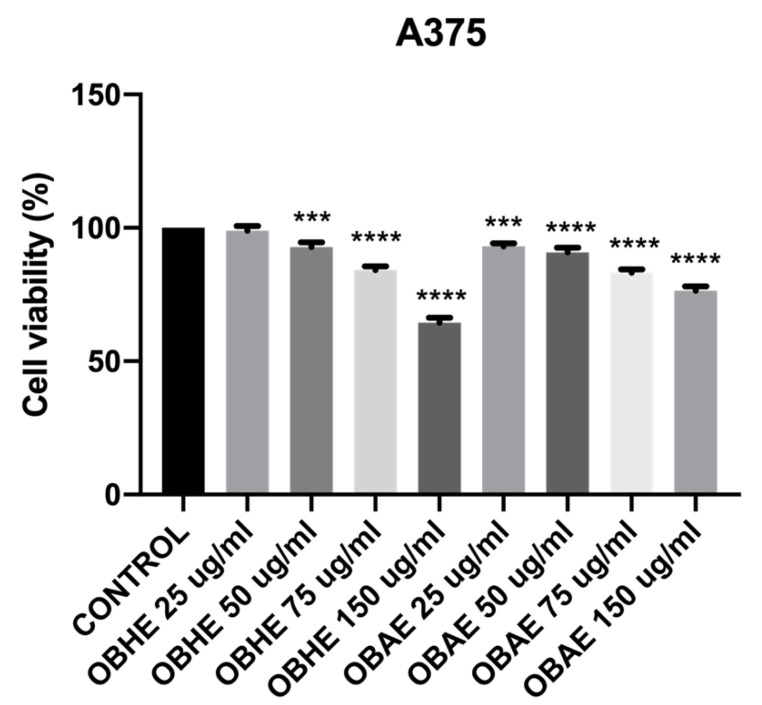
Viability percentage of human melanoma A375 cells after stimulation with basil hydro-alcoholic extracts (25, 50, 75 and 150 µg/mL), at 24 h post-stimulation. The results are expressed as cell viability percentage (%) normalized to control cell. The data represent the mean value ±SD obtained from three independent experiments performed in triplicate. One-way ANOVA analysis was applied to determine the statistical differences in rapport with positive control-treated cells followed by the Tukey’s test (*** *p* <0.001 and **** *p* <0.0001).

However, in the case of human melanoma A375 cells, the data obtained (Figure 3) revealed that both extracts, OBHE and OBAE manifest an important cytotoxic effect, the lowest viability (64.42%) being registered when the cells were exposed to the highest concentration—150 µg/mL of OBHE. Nevertheless, the A375 cells stimulated with the concentration of 150 µg/mL OBAE expressed viability of 76.40%. Apart from these treatment groups, cell viability rates were above 83%.

### 2.4. In Ovo Assessment of Hydro-Alcoholic Basil Extracts

**Figure 4:**
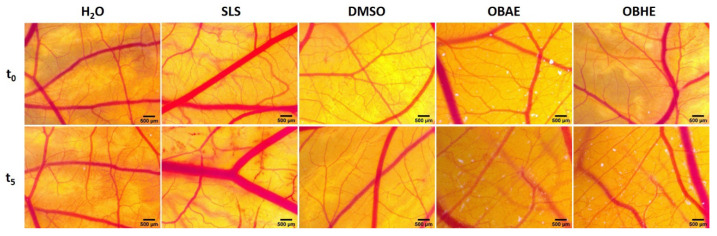
Irritability evaluation using the HET-CAM assay for basil hydro-alcoholic extracts (OBAE and OBHE, in concentration of 150 µg/mL) and control samples (water, SLS and DMSO 0.5%); images represent the CAM area of administration before sample application (**t_0_**) and five minutes after application (**t_5_**), by stereomicroscopy, magnification × 2.

The HET-CAM (hen’s egg chorioallantoic membrane) method was used to evaluate biocompatibility and to explore the potential irritability induced on irrigated mucosal tissues by hydro-alcoholic basil extracts. The assessment involved monitoring the potential haemorrhage, coagulability or blood vessel lysis when exposed to sample solution for the duration of 5 min. Both extracts tested at the highest concentration considered for this study (150 µg/mL) induced a very weak sign of irritation, according to the scale set by Luepke (Figure 4), with no severe alteration of the developing vascular plexus. The SLS sample used as positive control indicated a high degree of irritation, while distilled water, the negative control, had no irritative effect. The solvent used as dilution media of the extracts (DMSO, 0.5% in distilled water), showed a low IS value, thus being non-irritative.

Moreover, when comparing the IS values obtained for the two hydro-alcoholic basil extracts, 2.38 for OBHE, and 4.48 for OBAE (Table 4), the basil leaf extract showed a weaker irritation effect, close to the non-irritative potential.

### 2.5. Assessment of Skin Biophysical Parameters after Treatment with Hydro-Alcoholic Basil Extracts

**Figure 5:**
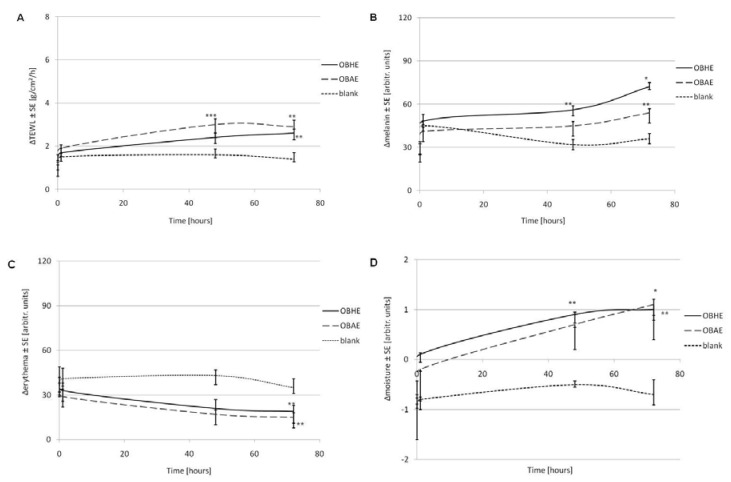
Changes of skin parameters in time: (**A**)—transe(pidermal water loss, (**B**)—melanin, (**C**)—erythema, and (**D**)—moisture of stratum corneum (* *p* < 0.05; ** *p* < 0.001; *** *p* < 0.001).

In order to verify the possible modifications of the skin parameters after exposure to the hydro-alcoholic basil extracts, in vivo evaluation was performed on healthy human skin. As presented in Figure 5, no important modification of the main skin parameters was found during these tests: the level of the transepidermal water loss (TEWL) has changed with just around 2.5 g/m^2^/h in the case of both extracts while the modification was approx. 1.5 units for the reference; the melanin has increased with around 60 units for both extracts and 30 units for reference, but it is important to know that its scale is large, ranging between 0 and 999 units.

**Figure 6:**
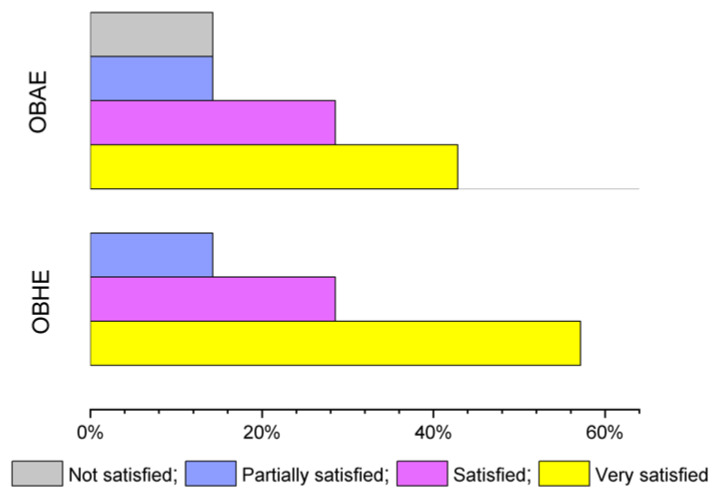
Satisfaction score for test basil extracts, based on leaves (OBHE) and aerial part (without leaves—OBAE), after three days’ test.

At the end of the study, the female volunteers were asked to evaluate the overall satisfaction regarding the sensation given by both hydro-alcoholic extracts on skin, by using the following score: 0—not satisfied, 1—partially satisfied, 2—satisfied, and 3—very satisfied, respectively. As shown in Figure 6, the satisfaction scores of OBHE hydro-alcoholic extract were higher than the OBAE hydro-alcoholic extract (57.14% vs. 42.85%, *p* < 0.05). Following applications of both hydro-alcoholic basil extracts, no adverse reactions were observed.

## 3. Discussion

According to the World Health Organization, the vast majority of the population living in developed countries place great emphasis on the use of herbs for the treatment of primary health condition [25]. Researchers in the whole world are concerned regarding the investigation of the pharmacological effects of plants, due to the fact that these effects are not yet known for all plant species. It is estimated that only a quarter of existing plant species have already been investigated [26]. The rich phytochemical composition of the plant the greater pharmacological effect. Antimicrobial, anti-inflammatory and antioxidant are common activities which have been identified at many plant species [27].

*Ocimum basilicum* L. (basil) is an ancient plant used in traditional medicine due to its therapeutic potentials. In certain regions of the world, the fruits of basil are used against worm infestation, inflammations, combating diarrhea, even to treat certain eye diseases. People of Vietnam regularly consume basil infusions, due to its anthelmintic, diaphoretic, antiemetic, and antidiarrhoeaic properties. If they are well separated and prepared, parts of basil could be successfully used against pains, infections, impotency, hypertension and diabetes [28]. Phenolic profile screening of *Ocimum basilicum* L. hydro-alcoholic extracts reveal a rich content regarding the investigated parameters (phenols, flavonoids, flavonols and condensed tannins), higher in the case of *Ocimum basilicum* L. hydro-alcoholic extract obtained from leaves (OBHE) than that obtained from aerial parts (OBAE) (Table 1). In a research study, Vlase and co-workers have determined the phenolic profile of *Ocimum basilicum* L. ethanolic extract [29]. Comparing the results reported by the authors, our results are different and have shown increased activity. The authors found a total phenolic content of 175.57 ± 2.43 mg GAE/g plant material in ethanolic extract of *Ocimum basilicum* L. and a total flavonoids content of 6.72 ± 0.19 mg RE/g plant material. Furthermore, the results reported in the present study exceeded the results obtain for other samples of *Ocimum basilicum* L. from Romania using various extractions emphasized. As an example, Benedec and co-workers [30] obtained a value for total phenolic compounds equal with 7.36 ± 0.19 g RAE/100 g plant material. The values obtained in the present study regarding the total phenolic content and flavonoid content also exceeded the results obtain for aqueous and ethanolic extracts of *Ocimum basilicum* L. obtained by Yesiloglu and Sit [31]. The authors have reported that the values for the aqueous extract were: TP = 100 mg GAE/g extract and TF = 42.5 mg CE/g extract, whereas the values for the ethanolic extract were: TP = 116 mg GAE/g extract and TF = 24.2 mg CE/g extract. The ethanolic extract of *Ocimum basilicum* L. prepared by Rezzoug and co-workers [32] had a slightly lower phenolic compound content (226 mg/g, gallic acid equivalents) as against our results (231.62 mg GAE/g dm) and the highest flavonoid content (213 mg/g, rutin equivalents).

Regarding the total condensed tannins (TT), our results revealed again that in the hydro-alcoholic extract obtained from leaves of *Ocimum basilicum* L., it was determined the highest content of tannins—11.18%, as against hydro-alcoholic extract from aerial part of basil in which was found it a quantity equal with 8.27%. The values of the present findings are much higher than the values reported by Elansary and Mahmoud [33]. They found that the total condensed tannins for a tea infusion of *Ocimum basilicum* L. leaves was 0.4 mg/g extract.

For the identification of the individual phenolic compounds in both hydro-alcoholic extracts, the retention times and mass spectral were compared with the calibration standards. By using the external calibration method, which is based on peak areas, all quantification was performed. The LC-MS analysis revealed the presence of nine phytocompounds in both hydro-alcoholic extracts, such as: gallic acid, protocatechuic acid, caffeic acid, epicathechin, coumaric acid, rutin, resveratrol, quercetin and kaempferol (Table 2). Many research studies have focused on exhibiting the pharmacological activities of these phytocompounds, which were also found by our research group in both hydro-alcoholic basil extracts investigated. In the literature data, these bioactive phytocompounds types, like—flavonoids, phenolic acids, and alkaloids have been shown to exhibit anti-inflammatory, antitumor, antioxidant, antimicrobial, antidiarrhoeal, antidepressant, hepatoprotective and anxiolytic properties. Moreover, both hydro-alcoholic basil extracts investigated in the present study are rich in flavonoids, flavonols, phenols, and tannins, which are polyphenolic compounds with demonstrated biologically activity against free radicals, microorganisms, toxins from liver, tumors and inflammation [34]. Bihari et al. [35] investigated the aerial parts of *Ocimum basilicum* L. and found the presence of flavonoids, phenolic compounds, protein, aminoacids, glycoside, gums, mucilage, tannins, flavones, triterpenes, steroids, and saponin. In a study conducted by Rezzoug and co-workers [32], the HPLC analysis of an ethanolic extract obtained from aerial parts (leaves) of *Ocimum basilicum* L. detected 15 phenolic compounds. The data revealed that the ethanolic extract of *Ocimum basilicum* L. had high amounts of rutin, epicatechin and vanillic acid: 476.28 μg/g, 225.01 μg/g, and 138.24 μg/g, respectively. Two phenolic compounds, quercetin (0.36 μg/g) and caffeic acid (6.48 μg/g), were found in low concentrations. By comparing their results with ours, it could be concluded that the differences might have come from the extract preparation and from the solvent used; since Rezzoug’s group used ethanol absolute as the extraction solvent, whereas in our case, 70% ethanol was used. Moreover, Vlase and co-workers [29] prepared an ethanolic extract from all the aerial part of *Ocimum basilicum* L. identifying and quantifying two hydroxycinnamic acid derivates (namely ferulic acid and p-coumaric acid); three flavonoid glycosides, isoquercitrin (quercetin 3-glucoside), rutin (quercetin-3-*O*-rutinoside) and quercitrin (quercetin 3-rhamnoside) and two free flavonoid aglycons (quercetin and luteolin), found in small quantities. Caftaric, gentisic, caffeic and chlorogenic acids were also identified in the basil extract, but they were in too low concentrations to be quantified. Kwee and Niemeyer [36] reported that the methanolic extract of basil contains individual phenolic compounds, influencing its antioxidant capacity. However, regarding the major differences recorded between our research group and other researchers in terms of LC-MS analysis’ results, they could have come from various parameters such as: extraction method applied, extraction solvent, extraction time and so on [37].

Nevertheless, in the present study, we demonstrate the presence of bioactive phytocompounds in both hydro-alcoholic extracts of *Ocimum basilicum* L. This indicates that both extracts could be successfully exploited for the prevention of various illnesses, such as wound healing, infections, inflammatory, and other conditions associated with oxidative stress.

Nowadays, the researchers have paid more attention to the antioxidant products from natural sources [38]. The evaluation of the antioxidant activity is directly related to type and plant organs; geographic region; conditions of the atmosphere; soil consistency; as well as to the method employed for antioxidant activity evaluation. Many methods regarding the assessment of AOA, both in vitro and in vivo, are published in literature. The DPPH assay is by far the most widely used method due to its simplicity, low cost, reproducibility and precise result. DPPH method is based on the ability of an antioxidant (contained in the plant extract) to reduce an oxidant (in this case—DPPH radical). The redox reaction takes place with color change, from deep purple in solution to pale yellow. The stronger the oxidant is decolorized, the higher the antioxidant capacity is [39]. In order to understand and elucidate various modes of action of antioxidants, a single method applied is insufficient [39].

In this study, for the antioxidant activity assessment of the two types of *Ocimum basilicum* L. hydro-alcoholic extracts, the DPPH method was employed. Our results revealed the fact that in the case of hydro-alcoholic extract obtained from *Ocimum basilicum* L. leaves, the inhibition percent obtained for scavenging the DPPH radical was almost comparable with the standard reference, meaning vitamin C (Figure 1). Vitamin C was chosen as standard reference, due to the fact that ascorbic acid is a very potent reducing agent and a well-known scavenger of free reactive radicals. In a mixture, vitamin C reacts with free reactive radicals (or donate electron), thus totally reducing their reactivity. Both hydro-alcoholic extracts have shown high antioxidant activity (68% in the case of OBAE extract at 100 µg/mL and 89% in the case of OBHE extract at the same concentration). Following the evaluation of several concentrations of both hydro-alcoholic extracts, the results showed that both hydro-alcoholic extracts of *Ocimum basilicum* L. demonstrated a significant antioxidant activity in a concentration-dose dependent manner (Table 3). These data are in accordance with the literature [33,40].

Corroborating the results obtained regarding the phenolic profile with those obtained in the case of antioxidant activity, we can affirm that there is a strong relationship between the concentrations of phenolic compounds in hydro-alcoholic extracts and their free radical scavenging activity. The presence of a higher content of phenolic compounds in hydro-alcoholic extracts leads to the improvement of their antioxidant potential. An improved antioxidant potential shows that *Ocimum basilicum* L. hydro-alcoholic extracts may be subservient to pharmaceuticals industry, with the purpose of formulating a drug/nutritional supplement, which may help in the prevention or treatment of various problems that could arise through the formation of reactive oxygen species (ROS) [41]. In order to increase the effective inhibition of ROS, the production of phytocompounds must be increased. To accelerate the biosynthesis of secondary metabolites, the plant cells could be expose to ultraviolet radiation which act as an abiotic component, thus allowing the plant’s defense mechanism to produce and stimulate the essential phytochemicals [42,43]. In a study conducted by Nazir et al. [44], the in vitro cell-free antioxidant potential of *O. basilicum* extract was evaluated using yeast cells. In order to established a protocol of in vitro callus cultures of *O. basilicum*, the author’s culture the basil leaf on Murashige and Skoog medium augmented with different concentrations of plant growth regulators. When the basil leaf was treated with 2.5 mg/mL of α-naphthalene acetic acid regulator, the antioxidant activity evaluated by DPPH assay was 93%. Another recent study also led by Nazir et al. [45] describes the treatment of callus culture of *O. basilicum* with various concentrations of melatonin and UV-C radiations. Following the evaluation of in vitro antioxidant activity with DPPH test, the maximum value (90.6%) was observed when the callus culture of basil leaf was treated with UV-C for 50 min.

In brief, natural antioxidants act as defensive agents and protect human cells and the entire organism from pathogenic attack caused by ROS. The ROS causes oxidative damage on lipids, proteins, nucleic acids and other molecules, which can lead to various chronic health problems, such as Parkinson’s and Alzheimer’s diseases, cardiovascular diseases, aging, cancer and inflammatory diseases [46,47,48]. Nazir et al. [43] evaluated the production of ROS and nitrogen species (RNS), as well as the formation of lipid peroxidation, using two *Ocimum basilicum* callus extracts and DHR-123 as an indicator of ROS/RNS production. The results confirmed that both callus extracts based on basil have protective effect against oxidative stress, results confirmed also by the measurements of lipid membrane peroxidation. In another study [44], the same group of authors demonstrates that melatonin and UV-C are effective elicitors due to the perfect correlation between the induced production of phenolic compounds, and in vivo cellular antioxidant effect of *Ocimum basilicum* callus extracts. Thus, the phenolic compounds of basil with distinguished biological activities (antioxidant, larvicidal, antimicrobial, antileishmanial, antifungal and anticancer), exert beneficial effects on cells by protecting against oxidative damage, acting as protective shields against carcinogenesis.

Generally, the biological activity of a plant extract is directly proportional to the concentration of phytocompounds. A complex chemical composition ensures successful use of different type of plant extracts, essential oils, in the prevention or even cure of an increased number of infections, inflammatory and various conditions associated with oxidative stress, being very well correlated in the literature the reduction of ROS production with the biological profile of *Ocimum basilicum* L. In a recent study, Yoshikawa et al. [23] demonstrated that UVA irradiated human dermal fibroblasts (NHDFs cells) developed a low intracellular production of ROS and carbonylated proteins following treatment with a 50% hydro-ethanolic *Ocimum basilicum* extract, while Nazir et al. [49] reported a high inhibition of ROS and reactive nitrogen species (RNS) level of *Ocimum basilicum* by employing a plant in vitro biological system; the antioxidant potential being attributed mainly to two metabolites, rosmarinic acid and chicoric acid.

Oxidative stress can also lead to DNA damage, which increases the mutation rate of the cells thus carry on to an oncogenic transformation of them [50]. In addition, Storz [51] revealed that DNA damaging effects of ROS can activate intracellular signaling pathways and thus contribute to tumor development through the regulation of cellular proliferation, angiogenesis, and metastasis.

As a general observation, the results obtained in this study revealed that OBHE and OBAE induced only a slight dose-dependent cell viability reduction (Figure 2) on the healthy cell panel. Human melanoma A375 cells were affected only after treatment with the highest test concentration 150 µg/mL of OBHE and OBAE, the cells expressing viability of 64.42% and 76.42%, respectively (Figure 3). These results may be related to either cells’ phenotype or to the low concentrations used (25, 50, 75 and 150 µg/mL), since regarding the cytotoxicity of *Ocimum basilicum* L. on tumorigenic cell lines, Al-Ali and co-workers demonstrated that the cytotoxic effect was more pregnant when extract concentration equal with 160 and 320 µg/mL were used on the hormone-sensitive breast adenocarcinoma MCF-7 cell line, therefore, higher concentrations than those tested in the present study [52]. Nevertheless, taking into consideration these aspects, it would be recommended as a future research direction that cell viability assays to be repeated under the same parameters (hydro-alcoholic extracts and the cell lines) as those employed in the current study, but using higher concentrations (e.g., 250, 500, and 1000 µg/mL) to make a better correlation between the effect induced by higher concentrations (>250 µg/mL) of basil extracts on non-tumorigenic cells versus tumoral cell lines. In another study, Arshad Qamar et al. revealed that basil methanolic extract manifested anti-proliferative effects on MCF-7 cells, which can be related to the alterations of microtubule assembly induced by ursolic acid from the basil methanolic extract [53]. This group of authors fractionated the methanolic extract of *Ocimum basilicum* into petroleum ether in two fractions: soluble and insoluble. From the insoluble fraction four compounds were isolated, mainly ursolic acid, which was further applied at a concentration of 100 µM on MCF-7 cells to assess the cell viability percentage. Finally, we believe that the cytotoxic potential is given by the ursolic acid not by the basil methanol extract itself (like it was used in the present study). In addition, it is well-known that ursolic acid has antitumor potential; moreover, the authors sustained the fact that the anti-proliferative potential of *Ocimum basilicum* extract against breast cancer cells may be partly due to the effects of ursolic acid on F-actin and microtubules [53]. Kathirvel and Ravi demonstrated that *Ocimum basilicum* oil induced significant anti-tumor effect on other cell lines: human cervical cancer—HeLa cells and human laryngeal epithelial carcinoma—HEp-2 cells, showing an IC_50_ value of 90.5 and 96.3 mg/mL, respectively [54]. In this case, the authors used the MTT assay for cytotoxicity screening and found out that at concentrations between 150–300 µg/mL, the basil essential oil affects the percentage of cell viability significantly, reaching a rate of 100%. The essential oil of *Ocimum basilicum* showed a dose dependent cytotoxicity (from 20 to 300 µg/mL) on all cell lines tested. The same study reported a cytotoxic effect manifested by *Ocimum basilicum* oil on mouse embryonic fibroblasts—NIH 3T3 cells, still the IC_50_ value (120.7 mg/mL) is higher than those recorded for tumor cells [54].

Regarding the *Ocimum basilicum* extract bioactivity, several parameters can be optimized to lead to the extraction of more biologically active compounds with anti-tumor activity, such as: extraction method used by the authors, extraction time, temperature, solvent used, the compounds extracted and so on.

The Interagency Coordinating Committee on the Validation of Alternative Methods, together with 15 regulatory and research agencies, including Food and Drug Administration (FDA) began to focus, more than 10 years ago, on developing new promising alternative animal models, in order to reduce or even replace the use of animal models in in vivo studies. The promising alternative methods are designed both to reduce the number of animals used, thus exempting more animal models of pain and suffering, as well as to obtain preliminary data. One of the alternative methods proposed by the Interagency is the hen’s egg test on the chorioallantoic membrane (HET-CAM) [55]. The HET-CAM assay, an internationally validated method, is based on the evaluation of chorioallantoic membrane of embryonated hen’s egg damage in order to assess the potential irritation to the conjunctiva [56]. The potential irritancy was detected by observing adverse changes which occur in the CAM of the hen’s egg, after exposure to both hydro-alcoholic extracts obtained by aerial parts and leaves of *Ocimum basilicum* L. Correlating with the lack of severe cytotoxicity on skin-derived cell lines and on the in vivo skin measurements, the results obtained in the HET-CAM assay (Figure 4 and Table 4) confirms the biocompatibility and good tolerance of both basil hydro-alcoholic extracts on the vascular developing plexus. Comparing the outcome induced by the two extracts, the leaf extract showed a better degree of biocompatibility, causing minimal signs of irritability upon the vascularized chorioallantoic membrane. Other studies indicated *Ocimum basilicum* aqueous and alcoholic extracts as angiogenic, suitable for regenerative processes [57], while only much higher concentrations of 5 mg/mL induced antiangiogenic effects [58]. Thus low concentrations of basil leaf extracts might be beneficial in the wound healing process.

Tewameter, mexameter and corneometer are professional equipment, often used to assess changes of the skin biophysical aspects induced by topical phytochemical treatments that provide reliable data on transepidermal water loss, erythema index and skin hydration level [59,60]. The scientific literature reported increases in the TEWL parameter more than 7 g/cm^2^/h daily, erythema over 40 units and skin moisture over 4 units per day, to be specific to toxic agents [61]. However, the results obtained in skin tests (Figure 5), show no important increase of the TEWL parameter after treating the skin with both types of extracts. Nevertheless, since the decrease of the TEWL parameter is correlated with the improvement of the skin hydration level [60], it can be concluded that among test extracts, OBHE provides a better skin moisturizing effect compared to OBAE. The slight increased values of stratum corneum moisture recorded after 48 and 72 h represent a confirmation of the beneficial potential of these hydro-alcoholic extracts, as moisturized skin prevents premature aging [60]. In good agreement with these results, Rasul and Akhtar reported an anti-aging potential of an emulsion based on basil extract when applied topically in vivo to human skin [24].

Another important aspect observed following in vivo application of basil extracts is the erythema index which expresses a small decrease, probably due to the active ingredients of the extracts that seem to have a beneficial potential to heal skin lesions, as reported by other studies as well [62,63]. Collectively, these results suggest the anti-inflammatory and wound healing potential of the hydro-alcoholic basil extracts, OBHE and OBAE.

In good agreement with our results, other studies also reported the anti-inflammatory activity of *Ocimum basilicum* L. [64,65]. This effect could be explained by the suppressive activity exerted by *Ocimum* species on arachidonate metabolism, correlated with the inhibition of two enzymes involved in the process of inflammation, namely: lipoxygenase and cyclooxygenase [57]. Regarding the anti-inflammatory effect manifested by basil alcoholic extract, Selvakkumar et al. demonstrated the inhibitory activity of crude basil alcoholic extract on human peripheral blood mononuclear—PBMC cells, along with a decrease of specific pro-inflammatory markers, such as: tumor necrosis factor-α (TNF-α), interleukin-1β (IL-1β) and IL-2 [65]. Nevertheless, Pansanga and collaborators reported an inflammatory effect after the development of a microemulsion containing essential oils from *Ocimum basilicum*, showing that the product with 5% *Ocimum* essential oils induced irritation of human skin. However, the 3% product did not cause an inflammatory reaction and was considered safe for human use [66].

Besides its anti-inflammatory potential, *Ocimum basilicum* also presents other activity [60]. In a comprehensive review, Jadoon and co-workers discussed the anti-aging potential of several phyto-extracts from pharmaceutical products, where *Ocimum basilicum* was one of the selected botanicals due to its antioxidant properties. In addition, the same study reported a viscoelasticity of the facial skin, treated with an emulsion containing *Ocimum basilicum* ethanolic extract isolated from seeds [60]. Summarizing the literature data and the results obtained in this study, *Ocimum basilicum* L. could be considered a promising phytocompound for an anti-inflammatory, wound healing and anti-aging approach.

## 4. Materials and Methods

### 4.1. Materials

#### 4.1.1. Reagents and Culture Media

For the determination of the TP and for the antioxidant activity (AOA) assay, the following reagents were used: ethanol 96% (*v*/*v*) and Na_2_CO_3_ were purchased from Chemical Company SA (Iasi, Romania); gallic acid and 2,2-diphenyl-1-picrylhydrazyl (DPPH) (Batch No: #STBF5255V) were acquired from Sigma-Aldrich Company (Steinheim, Germany); the ascorbic acid was acquired from Lach-Ner Company (Prague, Czech Republic), and the Folin-Ciocâlteu reagent was attained from Merck (Darmstadt, Germany). For the determination of TF and TFv, NaNO_2_ purchased from Merck (Darmstadt, Germany) were used; AlCl_3_ 98% acquired from Roth (Karlsruhe, Germany) and NaOH pellets, acquired from Chim Reactiv SRL (Bucharest, Romania). (+)-Cathechin hydrate 98% used as standard for the determination of flavonoid content was acquired from Sigma-Aldrich Company (Steinheim, Germany). For the determination of TT, the phosphotungstic acid (PTA) 10% (H_3_PW_12_O_40_), hide powder and pyrogallol 98% (C_6_H_6_O_3_) were acquired from Sigma-Aldrich Company (Steinheim, Germany).

The specific media required for cell growth, such as: Dulbecco’s modified Eagle medium (DMEM), Eagle’s minimal essential medium (EMEM), and dermal cell basal medium were purchased from ATTC (LGC Standards GmbH, Wesel, Germany). Other specific reagents for cell culture—Trypsin/EDTA solution, phosphate buffer saline (PBS), antibiotic mixture, fetal calf serum (FCS) and Trypan blue solution were obtained from Sigma Aldrich (Munich, Germany), whereas the 3-(4,5-dimethylthiazol-2-yl)-2,5-diphenyltetrazolium bromide—MTT kit was purchased from Roche Diagnostics GmbH (Mannheim, Germany).

#### 4.1.2. Cell Lines

The cell lines utilized in the current research were: HaCaT—immortalized human keratinocytes (Code no 300493; CLS Cell Lines Service GmbH), 1BR3—human skin fibroblasts (cat. no. 90011801; European Collection of Authenticated Cell Cultures, Salisbury, UK), JB6 Cl 41-5a—newborn mice epidermis (CRL-2010TM, ATCC, LGC Standards GmbH, Wesel, Germany), HEMa—primary human epidermal melanocytes (PCS-200-013, ATCC, LGC Standards GmbH, Wesel, Germany) and one tumorigenic cell line—A375 skin primary achromic human melanoma cells (ATCC CRL-1619, LGC Standards GmbH, Wesel, Germany). All the cell lines were received as frozen items and stored in liquid nitrogen until the experimental utilization.

### 4.2. Methods

#### 4.2.1. Plant Extracts Preparation

*Ocimum basilicum* L. was received from Favisan Laboratories Lugoj, Romania, and the botanical identification of the plant was certified by Professor Dr. Diana Antal, at Pharmaceutical Botany Department, Faculty of Pharmacy, “Victor Babes” University of Medicine and Pharmacy Timisoara, and a voucher specimen with the number CD_05 is stored at the Herbarium of the faculty. Aerial part (without leaves) of *Ocimum basilicum* L. (OBAE) and leaves of *Ocimum basilicum* L. (OBHE) were extracted with ethanol 70% at room temperature for 7 days, away from light and moisture, according to the method described by Modaresi et al. [67], slightly modified. The final extracts were filtered, concentrated under reduced pressure at 35 °C using a rotary evaporator, subsequently lyophilized and stored in the refrigerator (−4 °C) until further testing. Both extracts—OBAE and OBHE—were characterized in order to enhance the knowledge about the phytochemistry of this ancient medicinal plant with important medicinal potential.

#### 4.2.2. Physicochemical Characterization of Plant Extracts

##### Total Phenolic Content Determination (TP)

The total phenolic content of both hydro-alcoholic extracts from basil leaves and aerial part was evaluated using the Folin-Ciocalteu method, slightly modified [68]. 0.2 mL of each sample (three replicates), 1 mL of 10% (*v*/*v*) Folin-Ciocalteu reagent and 0.8 mL of Na_2_CO_3_ (7.5%, *w*/*v*) were added and incubated at 25 °C for 30 min. The absorbance of each sample was measured at 760 nm using an UviLine 9400 Spectrophotometer from SI Analytics (Deutschland, Germany). An ethanolic solution of gallic acid was tested in parallel in order to obtain a calibration curve (R^2^ = 0.996). The total phenolic content of the two extracts were calculated as milligrams of gallic acid equivalents (GAE) per gram of dry material.

##### The Total Flavonoid and Flavonols (TF and TFv) Content

The TF and TFv content of both hydro-alcoholic extracts were evaluated using the aluminium colorimetric assay according to Rouphael et al. method, slightly modified [69]. Briefly, 0.5 mL of each extract was treated with 0.5 mL of AlCl_3_ ethanolic solution (2%), allowed to stand for 30 min at room temperature and for flavonols evaluation samples, 0.5 mL of each extract was mixed with AlCl_3_ 2% and CH_3_COONa 5%, and allowed to stand for 3 h at room temperature. Absorbance values were read at 417 nm for flavonoid content and at 445 nm for flavonols content by using the UviLine 9400 Spectrophotometer (from SI Analytics). Rutin was used as reference standard (R^2^ = 0.998) and results are expressed as milligrams of rutin equivalents (RE) per gram of dry material.

##### The Total Condensed Tannins Content (TT)

The TT content in both hydro-alcoholic extracts was determined by dosing polyphenols contained in the analyzed samples, which are adsorbed on the hide powder, according to the protocol described by our group of research in a previous study [70]. First of all, the filtrate of each hydro-alcoholic extracts was prepared by adding an exactly volume of water to a specific amount of each lyophilized extracts, and then, the mixture was heated in a water bath for 30 min. After cooling under running water, each mixture was quantitatively transferred to a 250 mL volumetric flask. The solution was filtered, and the first 50 mL of the filtrate was removed. Then, from each filtrate were calculated the total polyphenols and the polyphenols that were not adsorbed on the hide powder. After the pyrogallol solution was determined, the percentage content of tannins was expressed using the following Equation (1):

(1) $$TT\;=\;\frac{13.12\;\cdot \;\left(A_{1}\;-\;A_{2}\right)}{A_{3}\;\cdot \;m}$$

where: *TT*—tannins concentration of the analyzed samples (%, *m*/*m*), *A*_1_—the absorbance of the total polyphenols, *A*_2_—the absorbance of the polyphenols that were not adsorbed on the hide powder, *A*_3_—the pyrogallol solution absorbance and m—the weight of the lyophilized extracts.

##### Identification of Phenolic Compounds by Liquid Chromatography—Mass Spectrometry (LC-MS)

Both hydro-alcoholic basil extracts, prepared for LC-MS analysis, were homogenized with a WisdVM-10 vortex mixer (Witeg Labortechnik, Wertheim, Baden-Württemberg, Germany) and centrifuged for 2 min at 10.000 rpm in a ThermoMicro CL17 micro-centrifuge (Thermo Fisher Scientific, MA, USA). The supernatant was collected and submitted to LC-MS analysis on a Shimadzu (2010 EV, Kyoto, Japan) chromatograph, which comprises an LC unit with a UV-VIS spectrophotometer detector (SPD-10A, Shimadzu Europe, Duisburg,), connected in-line with a MS-2010 mass spectrometer. Specific quantitative analysis of individual phenolic phytocompounds was performed on an EC 150/2 NUCLEODUR C18 Gravity SB 150 mm × 2.0 mm column, particle size 5 μm (Macherey-Nagel GmbH & Co. KG, Düren, Germany) operating at room temperature. The compounds were separated with gradient elution of two mobile phases A (aqueous formic acid) and B (acetonitrile and formic acid). The gradient program was: 5% B (0–20 min); 40% B (20–50 min) and 95% B (50–60 min).

#### 4.2.3. Antioxidant Activity (AOA) Evaluation

The antioxidant activity (AOA) of both hydro-alcoholic basil extracts was determined by DPPH (2,2-diphenyl-1-picrylhydrazyl) free-radical scavenging assay, according to the method of Miliauska et al. [71], modified by our research group. Thereby, it was prepared a fresh solution of DPPH in ethanol (1 × 10^−4^ M) and an amount equal with 1 mL of this solution was added to 3 mL of hydro-alcoholic basil extracts, at different concentrations (100 μg/mL; 50 μg/mL and 25 μg/mL). For 20 min, the absorbance of each concentration of basil extracts was measured in a continuously mode at 517 nm, using a UviLine 9400 Spectrophotometer from SI Analytics. The decrease of absorbance in the reaction mixture (*A_sample_*) indicates higher free radical scavenging activity. A blank sample consisting of 3 mL of hydro-alcoholic solution in 1 mL DPPH freshly prepared was measured at the same wavelength (*A_DPPH_*) for 20 min. The experiment was carried out in triplicate, using ascorbic acid in ethanol solution (100 µg/mL) as standard reference, positive control.

The DPPH radical scavenging capacity was calculated using the following Equation (2):

(2) $$\mathrm{DPPH}{\;}_{\mathrm{scavenging}\;\mathrm{effect}}\;\left[\%\right]\;=\;100\;-\;\frac{A_{sample}}{A_{DPPH}}\;\cdot \;100$$

#### 4.2.4. Cell Culture Conditions

The cells were cultured in specific growth media, as follows: HaCaT and A375 cells in DMEM, supplemented with 10% fetal calf serum (FCS), 1BR3 cells in EMEM supplemented with 15% FBS, JB6 Cl 41-5a in EMEM supplemented with 0.1% non-essential amino acids and 5% FCS and HEMa cells in dermal cell basal medium supplemented with adult melanocyte growth kit. An antibiotic mixture of 100 UI/mL penicillin and 100 μg/mL streptomycin was added to all cell cultures. All in vitro experiments were performed in sterile atmosphere using a biosafety cabinet class II (MSC Advantage 12 model (Thermo Fisher Scientific, Inc., Waltham, MA, USA). The cells were kept in standard conditions—humidified atmosphere with 5% CO_2_ at 37 °C by using a Forma™ Steri-Cycle™ i160 CO_2_ Incubator (Thermo Fisher Scientific, Inc., Waltham, MA, USA). Cell counting was performed by the means of the Countess II FL Automated Cell Counter device (Thermo Fisher Scientific, Inc., Waltham, MA, USA) in the presence of Trypan blue.

#### 4.2.5. Cell Viability Assessment by the Means of 3-(4,5-dimethylthiazol-2-yl)-2,5-diphenyltetrazolium Bromide (MTT) Assay 

The MTT assay was employed to evaluate the cell viability of all five selected cell lines (normal—HaCaT, JB6 Cl 41-5a, 1BR3, HEMa and and tumorigenic—A375 cells) after 24 h post-treatment with different concentrations (25, 50, 75, and 150 µg/mL) of OBAE and OBHE. This technique is a consecrated method used to evaluate the cell viability of various cell lines exposed to diverse compounds, including phytocompounds. In brief, 1×10^4^ cells/well were plated onto a 96-well plate. The following day, cell media was removed and the cells were treated with different concentrations of OBAE and OBHE (25, 50, 75, and 150 µg/mL) for 24 h. Control cells were treated with specific growth media. To determine cell viability rate, the absorbance of each well was measured at 570 nm wavelength, by the means of spectrophotometry, using a microplate reader (xMarkTM Microplate; Bio-Rad Laboratories, Inc., Hercules, CA, USA).

#### 4.2.6. HET-CAM Assay

The in vivo hen’s egg chorioallantoic membrane test (HET-CAM) was performed in order to assess the irritation potential and biocompatibility of these extracts on skin and mucosal tissues. The chick embryo chorioallantoic membrane from fertilized eggs was prepared following the standard CAM assay protocol [72], and the HET-CAM assay was performed as indicated by the ICCVAM recommendations (Interagency Coordinating Committee on the Validation of Alternative Methods [73,74]) with slight modifications.

Test samples (OBAE and OBHE dissolved in dimethyl sulfoxide (DMSO) 0.5% in distillate water), next to positive control (sodium lauryl sulphate (SLS) 0.5% in distillate water), negative control (distillate water) and solvent control (DMSO 0.5%) were inoculated onto the developing CAM in volumes of 300 µL. The application was performed on the 9 embryonic day of development, under stereomicroscopic (Discovery 8 Stereomicroscope, Zeiss, Göttingen, Germany) observation, capturing significant images initially, before application (t_0_) and 5 min after application (300 s, t_5_) of the CAM surface using the attached camera (Axio CAM 105 color, Zeiss, Göttingen, Germany) All captures were then processed using Zeiss ZEN software (Carl Zeiss Microscopy GmbH, Göttingen, Germany; https://www.zeiss.com/microscopy/int/products/microscope-software.html), Gimp 2.8 and ImageJ software (U.S. National Institutes of Health, Bethesda, MD, USA; https://www.gimp.org/ -for Gimp 2.8 and https://imagej.nih.gov/ij/index.html -for ImageJ).

The experimental procedure involves observing for 300 s the live modifications that are occurring after sample application in terms of hemorrhage—H (blood vessel bleeding), vascular lysis—L (disintegration of blood vessels), coagulation—C (intra or extra-vascular protein denaturizing), noting for each parameter the time (in seconds) that the first event appeared. Subsequently an irritation score (IS) was calculated using the following formula:

(3) $$IS\;=\;5\;\times \;\frac{301\;-\;Sec\;H}{300}\;+\;7\;\times \;\frac{301\;-\;Sec\;L}{300}\;+\;9\;\times \;\frac{301\;-\;Sec\;C}{300}$$

where: H = haemorrhage; L = vessel lysis; C = coagulation; Haemorrhage time (*Sec H*) = onset of haemorrhage reactions on CAM (in seconds); Lysis time (*Sec L*) = onset of vessel lysis on CAM (in seconds); Coagulation time (*Sec C*) = onset of coagulation formation on CAM (in seconds). Means values are obtained. The IS values range on Luepke scale between 0 and 21 as follows: 0–0.9—non-irritant, 1–4.9 weak irritant, 5–8.9/9.9 moderate irritant and 8.9/9.9–21 strong irritant [75].

#### 4.2.7. Skin Biophysical Parameters Assessment

Examination of both hydro-alcoholic basil extract was done by non-invasive in vivo technique, measuring several bio-physical parameters of the skin. The evaluation of skin parameters was performed using a Multi-Probe Adapter (MPA) from Courage-Khazaka Electronic GmbH (Koln, Germany). Professional equipment, as Tewameter^®^ TM300 (MPA, Couragee-Khazaka Electronic GmbH, Koln, Germany) to investigate the transepidermal water loss (TEWL), Mexameter^®^ MX18 to determine the level of melanin and erythema, and Corneometer^®^ CM825 (MPA, Couragee-Khazaka Electronic GmbH, Koln, Germany) for detection of skin hydratation. Seven healthy human volunteers (women; 23.5 ± 4.1 years old) were subjected to the examination for a 72 h patch-test, after obtaining the ethical approvement of “Victor Babes” University of Medicine and Pharmacy Timisoara—Faculty of Pharmacy Committee no2/2019 and respected all the requirements for tests of non-toxic natural compound on human skin, according to the Helsinki Declaration on Human Rights. All the in vivo evaluations were performed under the coordination of a dermatologist (Dr. Andrada Iftode).

Young volunteers were selected based on the fact that their skin is more likely not to have undergone physiological aspects that could interfere with the biophysical parameters of the skin, assessed in the present study; considering that mature skin is more prone to skin barrier impairment compared to young skin. Moreover, younger skin is more sensitive than mature skin [76], thus providing adequate conditions for quantifying erythema index. At the selection of the volunteers, it was also taking into account the exclusion criteria which refer to: the absence of skin-related pathologies and/or abnormalities (contact dermatitis, psoriasis, eczema, acnes, etc.), acute and/or chronic inflammation, allergy to the tested compound and skin infection. The female volunteers were informed that they are not allowed to expose themselves to the sun or artificial UV rays within 3 days and pregnant women and those who are breastfeeding were excluded from the study.

The concentration of both extracts (OBAE and OBHE) was determined by weighing the lyophilizate obtained at the end of the synthesis process, followed by its sonication using bi-distilled water as solvent and obtaining a concentrate mixture of 1 mg/mL.

Thereafter, both extracts were applied on different areas of their forearms and a sample containing just the solvents was used as reference. Their forearm skin of both hands was under occlusion for 72 h. The left forearm was treated with the OBAE extract and the right forearm was treated with OBHE extract and with the solvent. Values’ differences between after and before any treatment were expressed as mean and standard errors were calculated.

#### 4.2.8. Statistical Analysis

All the analyses were conducted in triplicate using Graph Pad Prism 7 and Origin 8 (Origin Lab—Data analysis and Graphing Software, Szeged, Ungary, https://www.originlab.com/2020b) for depiction and interpretation of the data. The results were expressed as average ±SD. Regarding the in vitro results, one-way ANOVA followed by the Tukey’s post-test was employed to establish the statistical differences between the different experimental and blank groups (* *p* <0.05, ** *p* <0.01, *** *p* <0.001 and **** *p* <0.0001).

## 5. Conclusions

The hydro-alcoholic extract of *Ocimum basilicum* L. obtained from leaves induced a significant antioxidant activity, the results being close to the value of ascorbic acid, used as standard reference. The results of the current study corroborate the traditional use of *Ocimum basilicum* L. as antioxidant and suggested that the high antioxidant effect was due to the high amount of total phenolic content and total flavonoids content obtained. Moreover, the results obtained from in vivo and skin biophysical parameters assessments indicated that *Ocimum basilicum* L. presents anti-inflammatory activity and promising wound healing properties. Both basil hydro-alcoholic extracts show a good biocompatibility and tolerance on the vascular developing plexus, as indicated by the HET-CAM assay. The hydro-alcoholic extracts applied in the range of concentrations tested seem to be optimal beneficial ingredients for wound healing and regenerative medicine and they may be included even in nutraceutical formulations if the plant extracts are characterized in terms of active compounds and biological profile. In addition, the in vitro evaluations have shown that *Ocinum basilicum* extract is a source of non-toxic active compounds on skin-related cells and it may act as a possible antimelanoma protective agent.

## Figures and Tables

**Table 1:** Total content of various phytocompunds classes from basil hydro-alcoholic extracts analyzed by spectrophotometric methods.

Basil Extract	Extract Yield (%)	TP (mg GAE/g dm)	TF (mg RE/g dm)	TFv (mg RE/g dm)	TT (%)
**OBAE**	17.2	177.36 ± 2.65	26.58 ± 1.33	2.39 ± 0.46	8.27 ± 2.86
**OBHE**	20.3	231.62 ± 0.42	48.22 ± 2.05	9.43 ± 1.51	11.18 ± 1.69

Legend: OBAE—*Ocimum Basilicum* L. aerial part hydro-alcoholic extract; OBHE—*Ocimum Basilicum* L. leaves hydro-alcoholic extract; TP—total phenolic content, TF—total flavonoids content, TFv—total flavonols content, TT—total condensed tannins. The results are expressed as average ±SD (*n* = 3).

**Table 2:** The main polyphenolic content of basil hydro-alcoholic extracts (OBAE and OBHE) analyzed by LC-MS.

Compound	Retention Time (min)	Conc. (µg/g) OBAE	Conc. (µg/g) OBHE
Gallic acid	5.123	2.134	4.206
Protocatechuic acid	11.506	0.638	0.020
Caffeic acid	18.150	0.046	0.148
Epicathechin	23.322	30.468	11.043
Coumaric acid	24.573	0.316	2.108
Rutin	25.615	4.684	24.869
Resveratrol	29.632	4.335	16.207
Quercetin	31.322	11.332	37.228
Kaempferol	34.391	40.085	1040.025

**Table 3:** The percent of inhibition antioxidant activity (AOA) induced by ascorbic acid as compared to *Ocimum basilicum* L. aerial part and leaves hydro-alcoholic extracts.

Ascorbic Acid		*Ocimum Basilicum* L. Aerial Part (OBAE)		*Ocimum Basilicum* L. Leaves (OBHE)	
Conc. [μg/mL]	% Inhibition	Conc. [μg/mL]	% Inhibition	Conc. [μg/mL]	% Inhibition
100	94.82 ± 0.052	100	67.51 ± 0.052	100	88.87 ± 0.045
50	50.17 ± 0.052	50	33.60 ± 0.014	50	60.52 ± 0.056
25	31.37 ± 0.045	25	21.77 ± 0.045	25	35.53 ± 0.052

**Table 4:** Irritation score induced by basil extracts. Values corresponding to the irritation score next to the irritation category are shown for the two basil extracts (OBEA and OBHE), positive (SLS 0.5%), negative (H_2_O) and solvent control (DMSO 0.5%).

Samples	Irritation Score (IS)	Irritation Category
H_2_O	0	No irritation
SLS 0.5%	18.7	Strong irritation
DMSO 0.5%	0.81	No irritation
OBAE 150 µg/mL	4.48	Weak irritation
OBHE 150 µg/mL	2.38	Weak irritation
